# Bacterial Adaptation by a Transposition Burst of an Invading IS Element

**DOI:** 10.1093/gbe/evab245

**Published:** 2021-11-13

**Authors:** Scott R Miller, Heidi E Abresch, Nikea J Ulrich, Emiko B Sano, Andrew H Demaree, Andrew R Oman, Arkadiy I Garber

**Affiliations:** Division of Biological Sciences, University of Montana, Missoula, Montana, USA

**Keywords:** transposable elements, insertion sequence elements, laboratory evolution, adaptation, clonal interference, *Acaryochloris*

## Abstract

The general importance of transposable elements (TEs) for adaptive evolution remains unclear. This in part reflects a poor understanding of the role of TEs for adaptation in nonmodel systems. Here, we investigated whether insertion sequence (IS) elements are a major source of beneficial mutations during 400 generations of laboratory evolution of the cyanobacterium *Acaryochloris marina* strain CCMEE 5410, which has experienced a recent or on-going IS element expansion and has among the highest transposase gene contents for a bacterial genome. Most mutations detected in the eight independent experimental populations were IS transposition events. Surprisingly, however, the majority of these involved the copy-and-paste activity of only a single copy of an unclassified element (ISAm1) that has recently invaded the strain CCMEE 5410 genome. ISAm1 transposition was largely responsible for the highly repeatable evolutionary dynamics observed among populations. Notably, this included mutations in multiple targets involved in the acquisition of inorganic carbon for photosynthesis that were exclusively due to ISAm1 activity. These mutations were associated with an increase in linear growth rate under conditions of reduced carbon availability but did not appear to impact fitness when carbon was readily available. Our study reveals that the activity of a single transposase can fuel adaptation for at least several hundred generations but may also potentially limit the rate of adaptation through clonal interference.

Significance

Bacteria exhibit great variation in the number of transposable elements (TEs) in their genomes; however, most of our knowledge of the impacts of TE activity on bacterial genome evolution is derived from model systems of limited phylogenetic diversity, so the importance of this variation for adaptation is not clear. Here, we show that the vast majority of beneficial mutations during laboratory evolution of *Acaryochloris marina* CCMEE 5410, which has among the highest transposase gene contents for a bacterial genome, are due to transposition of a single IS element. Our study has important implications for understanding the contribution of TEs to bacterial genome evolution.

## Introduction

The role of transposable elements (TEs) in adaptation continues to be debated. These mobile DNA sequences may confer a continuum of phenotypic effects on their hosts (Kidwell and Lisch 2001) but have often been considered to solely be genetic parasites (Doolittle and Sapienza 1980; Orgel and Crick 1980) with largely deleterious consequences for host fitness (Charlesworth et al. 1994). These include the disruption of gene regulation or function following transposition to a new location in the genome, large-scale genomic rearrangements resulting from ectopic recombination, and the generation of double-strand DNA breaks (reviewed by Nuzhdin 1999). More recently, however, investigations of insertion sequence (IS) elements—the simplest TEs, found in bacteria and archaea, which consist only of a transposase gene(s) encoding the mobilization machinery (Mahillon and Chandler 1998)—have concluded that a neutral model can explain observed patterns of IS distribution and abundance in bacterial genomes (Bichsel et al. 2013; Iranzo et al. 2014). Still, it is well-known that IS element activity can sometimes also be beneficial for the host through selectively favored null mutations, modified expression of adjacent genes, or large rearrangements (Hall 1999; Schneider and Lenski 2004; Gaffé et al. 2011; Hottes et al. 2013; Vandecraen et al. 2017).

Bacteria and archaea exhibit extensive natural variation in IS element number; most bacterial genomes contain no or few (<10) elements, whereas others have hundreds (Sawyer et al. 1987; Touchon and Rocha 2007; Bobay and Ochman 2017). There is also great variation within and between bacterial species in transposition activity and IS-mediated ectopic recombination rates (Nzabarushimana and Tang 2018). IS elements are predicted to contribute little to adaptive evolution when they are rare (Feher et al. 2012), but they can play a substantial role when moderately abundant. For example, during the initial stages (≤500 generations) of adaptation in *E. coli*, transposition or other structural variation involving IS elements (e.g., ectopic recombination) accounted for more than half of beneficial mutations for *E. coli* K12MG1655 (which has 44 TEs) evolved in the mouse gut (Barroso-Batista et al. 2014) and for ∼35% of mutations that attained high frequency in the Lenski long-term evolution experiment (LTEE; Deatherage et al. 2014; Consuegra et al. 2021; the ancestor clone REL606 has 49 TEs).

We therefore might expect that the relative importance of transposition for bacterial adaptation compared with other mutational mechanisms scales positively with IS element copy number and activity. However, little is known regarding whether these TEs are the predominant source of beneficial mutations for a microorganism with hundreds of IS elements in its genome. Similarly, we are largely ignorant of the potential constraints on adaptation that may be imposed by a high TE load due to, for example, clonal interference between competing adaptive mutations (Gerrish and Lenski 1998). In addition, most of our knowledge of the impacts of IS activity on bacterial genome evolution is derived from model systems of limited phylogenetic diversity (i.e., primarily proteobacteria). Consequently, expanding the scope of investigation to nonmodel organisms from different bacterial lineages is essential to provide more general insights on the role of IS elements for evolution.

To address these issues, we took a laboratory evolution approach with the cyanobacterium *Acaryochloris marina* strain CCMEE 5410, which was isolated from the Salton Sea, a moderately hypersaline lake in southern California (Wood et al. 2002; Miller et al. 2005). Strains of *A. marina* are unique in the production of Chlorophyll *d* as the primary photosynthetic pigment and have large genomes for bacteria, due in part to their high copy number of IS elements (Swingley et al. 2008; Miller et al. 2011). We report that the vast majority of selectively favored mutations during 400 generations of laboratory evolution involved IS element transposition and that most of these were due to the activity of a single IS element copy. This resulted in highly repeatable evolutionary dynamics among populations as well as clonal interference within populations, particularly at loci involved in inorganic carbon acquisition. The number of fixed IS transposition mutations was positively correlated with fitness during the linear phase of population growth, conditions under which carbon availability is expected to be limited.

## Results and Discussion

### Recent IS Transposition Burst in *A. marina* Strain CCMEE 5410

**Fig. 1.**
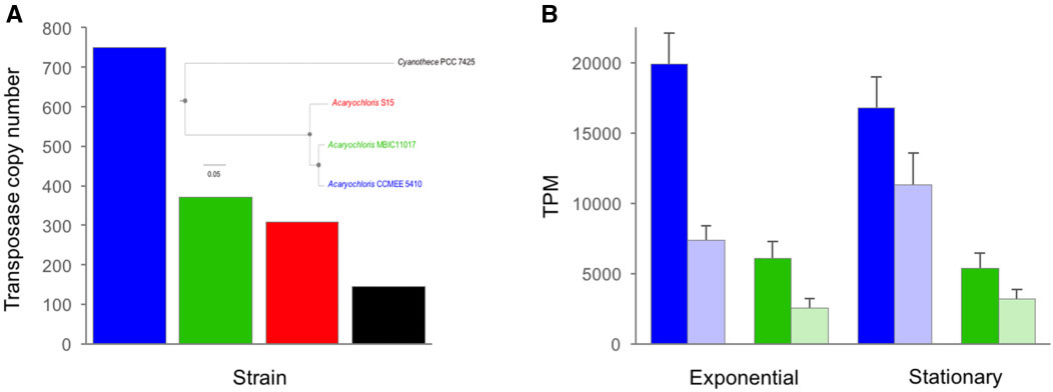
(A) Genome-wide number of transposase genes for *Acaryochloris* strains CCMEE 5410, MBIC11017 and S15 for the outgroup *Cyanothece* strain PCC 7425. *Inset:* Maximum-likelihood amino acid phylogeny of the four strains reconstructed from a concatenated alignment of 1468 orthologous proteins using a JTT+F+R5 substitution model. All nodes had 100% bootstrap support for 1,000 bootstrap replicates (indicated by closed circles). Scale bar is in units of expected number of amino acid substitutions per site. (*B*) Exponential growth and stationary phase expression (transcripts per kilobase million) of sense (dark shading) and antisense (light shading) transposase gene transcripts for *A. marina* strains CCMEE 5410 and MBIC11017. Error bars are standard deviations. Color coding as in (*A*).

We compared IS element copy number in the genomes of *A. marina* strains MBIC11017 (Swingley et al. 2008), CCMEE 5410 (Miller et al. 2011), and S15 (an epiphyte of the red alga *Pikea pinnata* isolated from Shelter Cove, CA in 2016), together with the outgroup strain *Cyanothece* strain PCC 7425. For this analysis, we used an improved assembly for *A. marina* strain CCMEE 5410 (NCBI BioProject ID PRJNA16707; 23 contigs, *N*_50_ = 4,516,345) and new genome data acquired for strain S15 (NCBI BioProject ID PRJNA649288; 7 contigs, *N*_50_ = 5,881,945). The CCMEE 5410 genome has a much greater number of IS elements compared with the other genomes (fig. 1*A*); in fact, it is an extreme outlier among bacterial genomes in general with respect to transposase gene content, both in terms of absolute number and percent of protein-coding genes (∼10%; supplementary fig. S1, Supplementary Material online). These include high element copy numbers for IS families that are either absent from or have a low copy number in the genome of sister taxon strain MBIC110017 (e.g., ISAs1; supplementary fig. S2 and table S1, Supplementary Material online). The differences among *A. marina* genomes cannot be explained by differences in genome size, which are comparable (8.09 Mb for CCMEE 5410 vs. 8.36 Mb for MBIC11017 and 7.11 Mb for S15).

Most IS elements in the CCMEE 5410 genome appear to be of recent origin based on the generally low levels of synonymous nucleotide divergence (d*S*) among duplicated gene copies within IS families (supplementary fig. S3*A*, Supplementary Material online); mean d*S* is among the lowest observed for a bacterial genome (supplementary fig. S3*B*, Supplementary Material online). This large number of identical or nearly identical full length copies of specific IS elements suggests on-going transposition activity. Still, many of the transposase genes of these elements have frameshifts and likely are pseudogenes (supplementary table S2, Supplementary Material online). Together, the above observations suggest that the high IS copy number in the *A. marina* CCMEE 5410 genome is the product of a recent or on-going expansion of IS elements from several IS families since it last shared a common ancestor with MBIC11017.

IS element expression comprised a disproportionately greater fraction of the CCMEE 5410 transcriptome compared with MBIC11017 than would be expected given the 2-fold difference in element number between the genomes (fig. 1*B*). This was the case for both sense and antisense transcripts and consisted of the expression of many different IS families (supplementary table S3, Supplementary Material online). In CCMEE 5410, ∼2% of sense transcripts were derived from IS elements during both exponential growth (mean ± SD = 2.0% ± 0.22%) and stationary phase (1.7% ± 0.22%), respectively. Because unnecessary gene expression is costly (Dekel and Alon 2005; Wagner 2005), we consequently expect IS expression to be a greater metabolic burden for CCMEE 5410.

### Major Role for Transposition of a Single IS Element during Laboratory Evolution

The *A. marina* CCMEE 5410 genome provides an excellent opportunity to address the consequences of a high TE load for evolution. To evaluate the relative contribution of IS activity to CCMEE 5410 evolution compared with other mutations, we conducted a laboratory evolution experiment with eight replicate populations derived from the same ancestral culture for which genome data were collected (see Materials and Methods). Experimental conditions were nearly identical to the strain’s recent culture history, with the exception of the culture volume (150 ml in 250 ml flasks during the experiment, compared with 50 ml in 125 ml flasks). After a lag, population growth under these batch culture conditions was characterized by a period of exponential growth followed by slower linear growth (supplementary fig. S4, Supplementary Material online). The transition from exponential to linear growth is commonly observed for cyanobacterial batch cultures and may be induced by light and/or carbon limitation as cell density increases (Sutherland et al. 1985; Schuurmans et al. 2017). Every 3 weeks (approximately seven generations), 1 ml of culture (∼450,000 cells) was transferred into fresh medium. The experimental populations were maintained in this way for 400 generations (∼40 months).

Every 100 generations we Illumina-sequenced DNA isolated from each population to greater than ∼30× coverage (supplementary table S4, Supplementary Material online) to identify new mutations. Because drift is expected to be weak compared with selection under these experimental conditions (*N_e_* is > 10^5^ in the evolving populations), mutations that rise to a detectable frequency in the population are likely either selectively favored or genetically linked to a beneficial mutation. Further, the observation of identical or parallel mutations at the same locus among populations constitutes strong evidence that the locus itself was the target of positive selection.

**Fig. 2.**
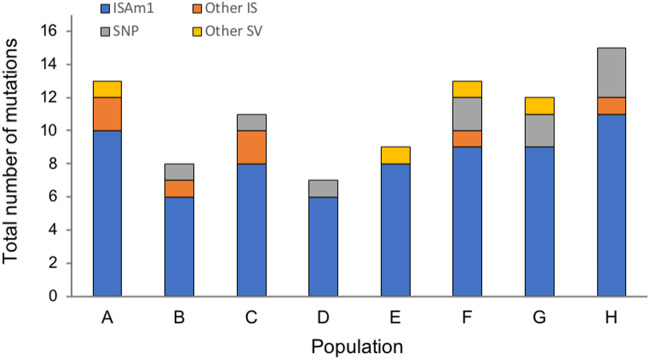
Distribution of mutations detected in the populations during the course of the experiment shows the massive contribution of ISAm1 insertions to laboratory evolution.

Most detected mutations (75–92% of mutations within each population, with a mutation frequency detection cutoff of 2%) were IS transposition events (fig. 2; for a complete description of detected mutations, including other kinds of structural variants and single-nucleotide polymorphisms, see supplementary table S5, Supplementary Material online). As predicted, this is a greater fraction than what has been previously observed in laboratory evolution experiments with *E. coli*, which has fewer IS elements (Barroso-Batista et al. 2014; Deatherage et al. 2014; Consuegra et al. 2021). We observed 39 distinct insertion alleles that were not found in the ancestor. Many of these were detected in multiple populations (supplementary table S5, Supplementary Material online) and were probably the result of convergent evolution (see below). Nearly two-thirds (*N* = 25) were in coding regions and are therefore likely null mutations, in accord with the idea that loss-of-function mutations can play an important role in adaptation (Hottes et al. 2013). Ten of these insertions had fixed (or were near fixation) by the end of the experiment (3,200 total generations of laboratory evolution, for about three fixed insertions per 1,000 total generations; supplementary table S5, Supplementary Material online). For comparison, among nonmutator lines in the LTEE, there were 355 fixed IS-mediated mutations over 658,500 total generations (∼0.5 per 1,000 generations; Consuegra et al. 2021).

Remarkably, however, this large contribution of IS-mediated mutations was not due to high TE load and activity per se. Rather, the overwhelming majority (≥80%) of these transposition events (and all that fixed) involved a single unclassified IS element (ISAm1) that consists of a single DDE transposase gene with a 14-bp inverted repeat (fig. 2 and supplementary table S5, Supplementary Material online). The direct repeats flanking the detected ISAm1 insertion sites have an average GC content of 27% (supplementary table S5, Supplementary Material online), suggesting a bias toward AT-rich sites (genome-wide GC content is 47.5% in coding regions vs. 41.5% in noncoding regions; supplementary fig. S5, Supplementary Material online). ISAm1 appears to have recently invaded the CCMEE 5410 genome, because it is not observed in the other *A. marina* strains. It is, however, homologous to a transposase gene from the cyanobacterium *Moorea* sp. (NCBI accession number NEP53674.1; 68% amino acid identity).

The genome of the CCMEE 5410 ancestor has nine nearly identical ISAm1 copies (supplementary fig. S6, Supplementary Material online). However, only one copy (genome coordinates 6:36060–6:37572) is complete; the others appear to be pseudogenes based on one or more premature stop codons resulting from frameshift mutations. Only the complete ISAm1 copy has 100% nucleotide identity with the reconstructed mRNA (supplementary fig. S6, Supplementary Material online), suggesting that it (and potentially its descendant copies) is the only transpositionally active copy; the other copies may be nonautonomous but possibly mobilized by this copy. ISAm1 transposition was by a copy-and-paste mechanism, and, at the end of the experiment, the number of ISAm1 copies segregating within populations had increased by 1–5 copies. In the ancestor, ISAm1 was transcribed throughout the batch growth cycle but exhibited highest expression (and highest ratio of sense vs. antisense transcripts) during lag phase (supplementary table S3, Supplementary Material online). By contrast, other IS elements were most transcriptionally active during other phases of the experimental growth cycle, for example, IS630 during exponential growth (supplementary table S3, Supplementary Material online). Consequently, the spectrum of IS-mediated mutations available to a bacterium may depend on its current or predominant physiological state (Maharjan and Ferenci 2017).

### Early Dynamics of Laboratory Evolution

**Fig. 3.**
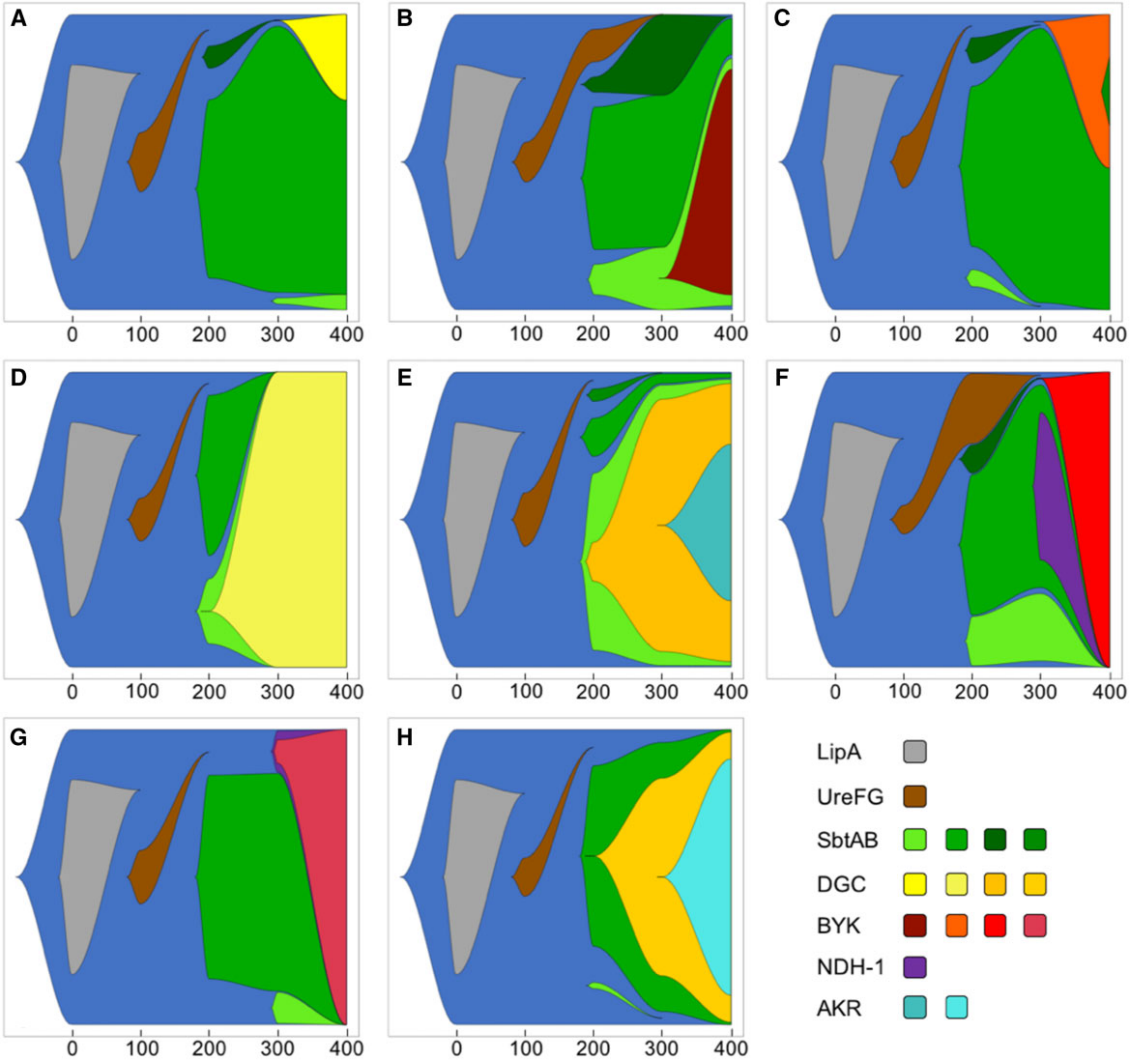
Fish plots of major evolutionary changes during 400 generations of laboratory evolution of the eight populations *A*–*G*. The majority of the selectively favored mutations shown are ISAm1 insertion events. The exceptions are DGC mutations in the *A* and *D* populations and BYK mutations in the *B*, *C*, and *F* populations.

Illumina sequencing of the ancestral population to ∼30× coverage (supplementary table S4, Supplementary Material online) revealed a few polymorphisms (including two low-frequency ISAm1 insertion polymorphisms; supplementary table S6, Supplementary Material online) that appear to have arisen during clonal outgrowth in preparation of cells for both genome sequencing and the initiation of the laboratory evolution experiment. All of this ancestral variation was eventually lost in all of the evolved populations, most by generation 100. After 100 generations, we also detected an identical ISAm1 insertion between the urease accessory protein coding genes *ureF* and *ureG* in all populations (fig. 3; for a complete list of detected mutations, see supplementary table S5, Supplementary Material online). This mutation was not detected in the ancestral population and may reflect an insertion hot spot, but we cannot rule out that it was segregating in the ancestral population at low frequency. This mutation was also lost in all populations later in the experiment as new beneficial mutations emerged (fig. 3).

### Repeatable ISAm1 Insertions in and near Inorganic Carbon Acquisition Genes

By generation 200, loci involved in inorganic carbon acquisition were among the most common and repeatable mutational targets that rose to a detectable frequency, and these mutations were exclusively due to ISAm1 transposition. For example, between one and three different ISAm1 transposition-mediated alleles were detected in the *sbtAB* operon in all populations (fig. 3 and supplementary table S5, Supplementary Material online). All three alleles were intergenic (a fourth ISAm1 insertion event in *sbtB* emerged in a single population late in the experiment), and two (Sbt-1 and Sbt-2) were observed in all populations. For multiple reasons, we believe that these mutations were convergent rather than standing variation. None of the alleles were observed prior to generation 200 (despite sequencing populations to greater than ∼250× coverage in generation 100; supplementary table S4, Supplementary Material online), yet one or more increased rapidly in frequency once detected (fig. 3 and supplementary fig. S7, Supplementary Material online). This suggests that they were under strong positive selection and would have been detected earlier in the experiment if they had been present in the ancestor. In addition, we would expect to have observed similar evolutionary trajectories across populations if they were derived from standing variation.

Together, *sbtA* and *sbtB* are involved in cellular acclimation to low carbon. In the CCMEE 5410 ancestor, *sbtAB* genes are coexpressed as a single ∼1.8-kb bicistronic transcript that is upregulated to 10-fold higher levels during carbon limitation (supplementary table S7, Supplementary Material online). SbtA is a sodium-dependent, high-affinity bicarbonate transporter that is a part of the cyanobacterial carbon-concentrating mechanism (Shibata et al. 2002), and SbtB is a P_II_-like cAMP-binding signaling protein that senses cellular energy status through adenylate binding and associates strongly with SbtA when bound to ADP or AMP (Selim et al. 2018; Förster et al. 2021). Although the consequences of SbtB interaction with SbtA remain to be fully resolved, multiple studies suggest that SbtB is a negative allosteric regulator that inactivates SbtA under conditions of low cellular adenylate energy charge (Du et al. 2014; Förster et al. 2021). *sbtB* inactivation does not impact bicarbonate uptake by SbtA in *Synechococcus* sp. PCC 7942 (Price 2011), and, in *Synechocystis* PCC 6803, cells of a *sbtB* deletion mutant appear to be acclimated to low carbon conditions (Selim et al. 2018). SbtB may also have a more general regulatory role in C_i_ acquisition beyond its direct interaction with SbtA: in the *Synechocystis* PCC 6803 *sbtB* deletion mutant, another bicarbonate transporter (*bicA*) that is also regulated by C_i_ availability in wild-type cells was constitutively expressed (Selim et al. 2018). These results suggest that SbtB deficiency may result in generally enhanced C_i_ assimilation, particularly under conditions of low C_i_ availability.

Two other distinct ISAm1 insertion mutations associated with C_i_ uptake were also detected in multiple populations at generation 200 or later, which indicates that they were independently acquired in the individual populations. These included identical insertions into a *sbtA* homolog (gene peg.7356, genome position 43:180977; 45% amino acid identity to SbtA and adjacent to a *sbtB* homolog) in seven of the populations (supplementary table S5, Supplementary Material online). This insertion was first detected at different times in different populations but never attained high frequency in any population (maximum observed frequency ranged from 9% to 27%). Potentially, selection was not strong on this mutation, because this gene is only lowly expressed under all conditions compared with, for example, *sbtAB* (supplementary table S7, Supplementary Material online). We also identified an ISAm1 insertion 85 nucleotides upstream of the NDH-1MS complex in three populations (position 0:83438; table S5, Supplementary Material online; fig. 3). This mutation had nearly swept through one population by the end of the experiment (fig. 3*G*) but had been lost in the other two populations as a result of sweeps by competing beneficial populations (fig. 3*D* and *F*). NDH-1MS is a cyanobacterial NAD(P)H: Quinone oxidoreductase complex specialized for high affinity CO_2_ uptake under low C_i_ conditions (Battchikova et al. 2011). Similar to what was previously reported for *Synechocystis* PCC 6803 (Zhang et al. 2004), ancestral CCMEE 5410 cells exhibited increased transcription of NDH-1MS genes in a low C_i_ environment, as did other carbon concentrating mechanism genes (supplementary table S7, Supplementary Material online).

### Resolution of Sbt Allele Clonal Interference often Involved ISAm1 Transposition

The emergence of multiple co-occurring Sbt alleles is expected to produce clonal interference dynamics (Gerrish and Lenski 1998), whereby competition between competing beneficial alleles slows the loss of variation from the population. Still, by the end of the experiment, Sbt diversity was lost in six of the eight populations (1–3 detected alleles vs. a maximum of 3–4 alleles), and a single allele had attained high frequency (fig. 3 and supplementary fig. S7, Supplementary Material online). Four of the five Sbt alleles became the majority allele in at least one population. This included the ancestral allele, which appeared to be generally selected against, because it was either undetectable or at a low frequency by the end of the experiment in most populations. However, in two populations (C, G; fig. 3), there was a substantial increase in the ancestral allele’s frequency between generations 300–400 as a result of new beneficial mutations that overcame this deleterious genetic background.

**Fig. 4.**
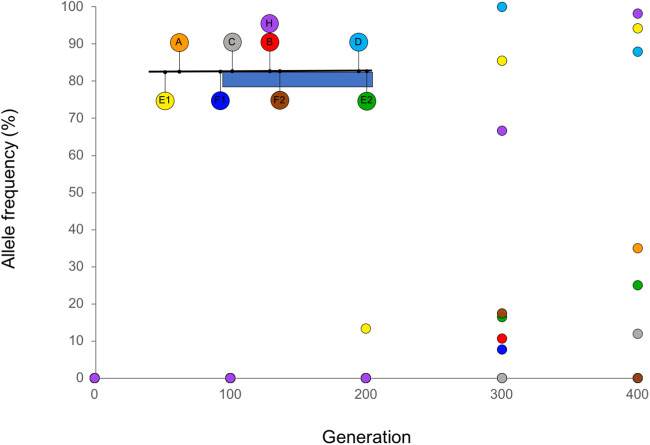
Location and frequencies of mutations detected during laboratory evolution in and near the annotated diguanylate cyclase gene peg.4655. Shown is a 1-kb region of the CCMEE 5410 genome (genome coordinates 0:4457436-0:44578436) including peg.4655 (blue rectangle) and upstream noncoding DNA. All mutations are IS transposition events, with the exception of the D allele, which is a nonsense mutation at amino acid position 207 (supplementary table S5, Supplementary Material online).

The evolutionary outcome of Sbt clonal interference was typically associated with beneficial mutations at one of two loci, half of which involved ISAm1 transposition. In three populations, sweeps of a particular Sbt allele (Sbt-1 in the D and E populations, Sbt-2 in H; fig. 3) were linked with mutations either within or upstream of a diguanylate cyclase gene (peg.4655; fig. 4 and supplementary table S5, Supplementary Material online). Mutations at this locus were very common following the emergence of Sbt variation: we observed a total of eight distinct alleles in seven of the populations (fig. 4), the majority of which interrupted the coding region and are therefore expected to be null mutations. Seven of the mutations were due to the transposition of IS elements (five by ISAm1 activity); by contrast, the D population allele, which was undetected in generation 200 but had been fixed in the population by generation 300, was a C-to-T mutation resulting in a premature stop codon. Therefore, although the majority of detected mutations involved IS transposition, other kinds of mutations also contributed to CCMEE 5410 adaptation during laboratory evolution.

Diguanylate cyclases are involved in the production of the secondary messenger molecule cyclic diguanylate, which activates specific effector proteins to impact a number of cellular processes, including biofilm formation and stress responses (Dahlstrom and O'Toole 2017). Evolutionary changes in cyclic diguanylate signaling have been previously shown to be central to diversification in biofilms of *Pseudomonas aeruginosa* (Flynn et al. 2016). In CCMEE 5410, peg.4655 is constitutively expressed (supplementary table S7, Supplementary Material online), and its ortholog in *A. marina* MBIC11017 is upregulated under microoxic conditions (Hernández-Prieto et al. 2017). Its effector protein and the downstream consequences of its inactivation remain to be determined.

In four populations, late-arising mutations in a bacterial tyrosine kinase (BYK) gene (peg.5255) had attained high frequency (53–100%) by the end of the experiment (fig. 3); these mutations arose on three different Sbt allele backgrounds, and two involved ISAm1 and ISAcma36 transposition events, respectively (the other three mutations were nonsynonymous SNPs; supplementary table S5, Supplementary Material online). BYKs are signaling proteins that regulate traits such as virulence, stress responses, and exopolysaccharide production by both autophosphorylation and substrate phosphorylation of tyrosine residues (Grangeasse et al. 2012). The insertions, which are located at sites eight nucleotides apart at the 3′ end of the gene, are expected to disrupt the C-terminal tyrosine cluster autophosphorylation sites of the protein. This could potentially disrupt interactions with its target substrate proteins. This gene also possesses a N-terminal GumC domain, which suggests that it is involved in exopolysaccharide biosynthesis.

### Faster Linear Growth of Evolved Populations Is Associated with the Number of Fixed ISAm1 Transposition Events

Dissection of the specific phenotypic effects of ISAm1-mediated mutations, as well as their underlying mechanisms, will be the subject of a future investigation. In particular, we propose that *sbtAB* insertions may enhance C_i_ acquisition through SbtB deficiency (see above). We can identify at least two ways in which C_i_ acquisition may have been under selection during laboratory evolution. First, our experimental treatment imposed a reduction in the ratio of gas exchange surface area to culture volume compared with the strain’s recent culture history. Therefore, environmental C_i_ availability is expected to be generally lower under the experimental conditions. C_i_ availability is also expected to fluctuate during the course of a growth cycle, with higher availability during early growth at low cell densities, followed by C-limitation later in the cycle.

**Fig. 5.**
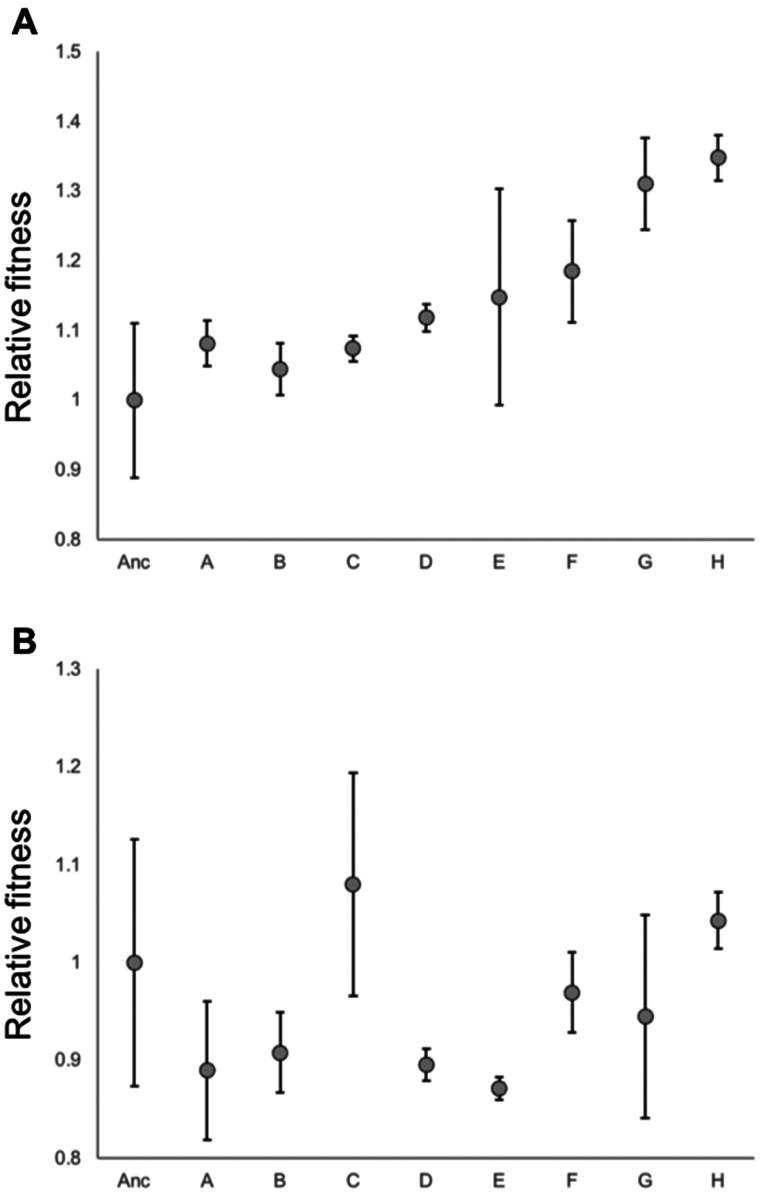
Relative growth parameter estimates for experimental populations after 400 generations of laboratory evolution, compared with the ancestral population (Anc): (*A*) linear growth rate, (*B*) exponential growth rate. Error bars are standard errors for triplicate independent cultures. The absolute values for the ancestral population were an increase in OD of 0.00135/h for linear growth and an exponential rate of OD increase of *e*^0.0163*t*^, respectively.

Consequently, we predicted that the evolved populations would grow faster than the ancestral population later in the cycle, when population growth is linear. This was indeed the case (fig. 5*A*; *t* = 1.78, *P* < 0.05 for a one-tailed test comparing the evolved populations vs. the ancestral population); by contrast, populations had not diverged in growth rate during exponential phase (fig. 5*B*; *t* = −0.63, *P* = 0.54). Although we did not have the power to distinguish statistically significant differences in linear growth rate between individual pairs of populations when corrected for multiple comparisons, we did observe that the estimated mean linear growth rates of the evolved populations were positively correlated with the number of fixed ISAm1 transposition events within populations (*R* = 0.78; *N* = 8; *F*_[1,6]_ = 9.19, *P* = 0.02). This suggests that fixed ISAm1 transposition mutations at C_i_ acquisition loci contributed to faster linear growth. It also suggests that clonal interference may have limited the rate of adaptation in the populations with the lowest estimated linear growth rates and for which no ISAm1 transposition mutations were fixed (populations A, B, and C).

### Impact of ISAm1 Activity and Its Long-Term Fate

We have shown that a single active copy of a TE can fuel the initial stages of adaptation over hundreds of generations of laboratory evolution. Transposition of the ISAm1 element was responsible for about 75% of beneficial mutations. These were the only IS-mediated mutations to sweep to fixation and did so rapidly, within 100–200 generations. This greatly increased the rate of adaptive mutations compared with nucleotide substitutions alone, as has been observed for an IS transposition burst during *E. coli* adaptation to a change in osmolarity (Stoebel and Dorman 2010).

Ancestral copy number for a given IS family does not necessarily predict transposition activity (Consuegra et al. 2021). Still, the predominance of a single TE for adaptation was striking in light of the fact that multiple IS families have high genome copy number and are actively expressed by *A. marina* CCMEE 5410 (supplementary fig. S2 and tables S1 and S3, Supplementary Material online). The reasons why we did not observe a more equitable contribution to adaptation from different IS families (including other recently acquired elements that are unlikely to have been domesticated) are not clear. Potentially, insertion site targets are more restricted or saturated for other highly expressed elements.

Although ISAm1 activity promoted short-term adaptation in our experiment, the long-term fate of the ISAm1 element is not clear. In the LTEE, mutations due to IS activity were also positively associated with fitness during the early stages of adaptation but constrained adaptation over the long term (Consuegra et al. 2021). Simulation studies of both sexual diploid and asexual populations have indicated that an invading TE is more likely to be stably maintained in a genome following an initial transposition burst if its activity is subsequently regulated (Wu et al. 2015; Le Rouzic and Capy 2005). Otherwise, it is ultimately expected to go extinct, provided that deleterious transpositions are much more common than adaptive insertions. In our experiment, beneficial ISAm1 transposition mutations with a large selective effect were sufficiently frequent to co-occur within a population (fig. 3), corresponding to a strong-selection strong-mutation regime (Gillespie 1991). However, we did not observe any compelling evidence for potentially deleterious ISAm1 transposition mutations hitchhiking to high frequency. Rather, the rare cases of multiple ISAm1 transposition events sweeping together were plausibly adaptive. For example, in the G population, there was a rapid sweep of three ISAm1 transposition events between generations 300 and 400 at loci that convergently rose to high frequency in other populations (bacterial tyrosine kinase, coproporphyrinogen III oxidase, and the NDH-1MS complex; fig. 3 and supplementary file 7, Supplementary Material online). Therefore, although beneficial ISAm1 transpositions were frequent enough to compete with each other, the probability of a deleterious transposition event hitchhiking along appears to be low. This suggests that deleterious transposition events may cause strong fitness effects and be effectively purged from the population, preventing the accumulation of a substantial deleterious ISAm1 load.

TE insertions have been shown to contribute to adaptation in natural populations of a variety of organisms, including *Arabidopsis thaliana* (Li et al. 2018), *Drosophila melanogaster* (González et al. 2008, 2010) and the peppered moth following the Industrial Revolution (van’t Hof et al. 2016). *Acaryochloris* *marina* is best known from shallow coastal environments, attached to red algae and marine invertebrates, and is expected to experience more heterogeneous and less predictable environments in nature than during laboratory evolution. Whether IS transposition also plays an important role in the adaptation of natural populations of *A. marina* is not known, as laboratory strain resources for this cyanobacterium are still limited (Ulrich et al. 2021). Future investigations will aim to address this issue with improved taxon sampling and a population genomics perspective on *A. marina* variation.

## Materials and Methods

### Laboratory Evolution Experiment

Cells of *A. marina* strain CCMEE 5410 derived from single colony selection on an agar plate were stored at −80 °C and later revived and grown at 30 °C in a 125-ml Erlenmeyer flask containing 50 ml of HEPES-buffered (10 mM final at pH 8.0) FeMBG-11 medium (IOBG-11 supplemented with iron(III) monosodium salt; Swingley et al. 2005). The culture was grown with constant shaking (92 rpm) on a VWR Advanced Digital Shaker and illuminated with 25 µmol m^−2^ s^−1^ of cool white fluorescent light on a 12 h:12 h light: dark cycle. Cells of this ancestral stock culture were grown to high density and used to establish eight replicate populations (A–H). Experimental populations were initiated by inoculating 1 ml each from the ancestral stock into 250 ml longneck flasks containing 150 ml of FeMBG-11/HEPES (10 mM final, pH 8.0) medium. Experimental medium, temperature, and light regime were identical to the ancestral maintenance conditions. Every 3 weeks (approximately seven generations), 1 ml of culture (∼450,000 cells) was transferred into 150 ml of fresh medium. Every 6 weeks, 25 ml of each population were collected prior to transfer, pelleted, and stored at −80 °C for DNA. Every ∼100 generations, samples were sent to the University of Pittsburgh Microbial Genome Sequencing Center for library construction and Illumina sequencing (see below).

### Genome Data and Analysis

Both short-read (Illumina) and long-read (PacBio) genome sequence data were acquired for *A. marina* strains CCMEE 5410 and S15. For CCMEE 5410, cells for Illumina sequencing were obtained directly from the ancestral stock culture used to inoculate the laboratory evolution population cultures (see above). For PacBio sequencing, 1 ml each of the ancestral population stock was inoculated into two flasks of FeMBG-11/HEPES and harvested after ∼10 generations of growth.

For Illumina sequencing, 120 µl of lysozyme (10 mg/ml) were added to a microfuge tube containing ∼0.1 g of pelleted culture. The tube was next vortexed and incubated at 37 °C for 30 min. Following this, DNA was extracted with the Qiagen DNeasy PowerBiofilm kit according to the manufacturer instructions. DNA was Qubit quantified and sent to the University of Pittsburgh Microbial Genome Sequencing Center for library preparation and 151-bp paired-end sequencing on an Illumina NextSeq 500 flow cell.

In addition, high-molecular weight DNA was extracted for PacBio sequencing from 100 ml of culture split into two pellets. Each pellet was resuspended in 4.7 ml of TE buffer (pH 8.0). We next added 100 µl of 200 mg/ml lysozyme to each tube and incubated at 37 °C for 45 min. Following this, 50 µl of Proteinase K were added, and the tubes were incubated at 55 °C for 1 h. 900 µl of 5 M NaCl were then added to each tube, followed by 750 µl of CTAB/NaCl (10 g cetyl trimethylammonium bromide and 4.09 g NaCl). After incubation at 65 °C for 20 min, cell debris was pelleted at 5,000 × g for 10 min at room temperature. The supernatant was transferred to a new tube to which an equal volume of chloroform was next added. The tube was then centrifuged at 5,000 × g for 30 min. Following this, the aqueous phase was harvested, and DNA was precipitated with 2× volume of 100% ethanol and then pelleted at 5,000 × g for 30 min. A total of 200 µl TE was added to dissolve the pellet, and the solution was transferred to a clean microfuge tube. A total of 200 µl of phenol/chloroform (1:1) was added to the tube, mixed well by repeated inversion, followed by centrifugation for 10 min at 17,000 × g. The aqueous layer was then transferred to a clean microfuge tube and extracted with chloroform an additional time as above. DNA was reprecipitated with ethanol as above, and then, after removing the supernatant, resuspended in 50 µl of 3 M sodium acetate (pH 5.2). We next added 10 µl of glycogen and 3.5× volume of 100% ethanol, followed by incubation at −80 °C for 30 min. The sample was then centrifuged at 17,000 × g and 4 °C for 15 min. Following this, the supernatant was removed, and the sample was air dried, resuspended in 10 mM Tris and stored at −80 °C. Sample quality was assessed with an Agilent Tapestation and by Qubit and Nanodrop. Sequencing was conducted with a PacBio Sequel System at the University of Maryland Institute for Genome Sciences. Genomes for *A. marina* strains CCMEE 5410 and S15 were de novo assembled with Canu v1.7 (Koren et al. 2017), and these assemblies were improved with Pilon (Walker et al. 2014) using Illumina data.

### Phylogenetic Analysis

Orthologous protein-coding genes were identified for *A. marina* strains MBIC11017 (GCA_000018105.1), CCMEE 5410, and S15 and for the outgroup strain *Cyanothece* PCC7425 (NCBI accession: GCA_000022045.1) using OrthoFinder v2.2.7 (Emms and Kelly 2019). A maximum-likelihood amino acid phylogeny with 1,000 ultrafast bootstrap replicates (Minh et al. 2013) was constructed with IQ-TREE v2.0 (Nguyen et al. 2015) using the JTT substitution matrix with empirical amino acid frequencies (+F) and five estimated free rate categories of rate heterogeneity among sites (+R5). The model was selected by the Akaike information criterion (AIC) with ModelFinder (Kalyaanamoorthy et al. 2017).

### IS Element Analyses

Genome-wide estimates of transposase gene copy number and IS family assignments were obtained by parsing annotation data with a custom Python script. Genomes were annotated using Prokka v1.14.6 (Seemann 2014), which uses the ISfinder database of ISs (Siguier et al. 2006) to identify transposases. Predicted proteins were queried against the ISfinder database using BLAST, with an *e*-value cutoff of 1E-9. The transposase family affiliation is included in the Prokka output, and this information was also extracted from the general feature format (GFF) file that is included among Prokka’s output files. To identify which transposase genes were related by gene duplication and to measure the amounts of synonymous and nonsynonymous nucleotide divergence between pairs of transposase duplicates, we developed a novel bioinformatics software, ParaHunter, which is freely available on GitHub: https://github.com/Arkadiy-Garber/ParaHunter (last accessed December 7, 2020). ParaHunter identifies homologs by clustering genes using *mmseqs2* v6.f5a1c (Steinegger and Söding 2017), based on user-chosen parameters of minimum amino acid identity and coverage. After gene clusters are identified, each cluster is aligned using *Muscle* v3.8.1551 (Edgar 2004). ParaHunter then uses *codemL* in PAML (Yang 1997) to generate codon alignments (*pal2nal.pl*) and estimate rates of synonymous (*dS*) and nonsynonymous (*dN*) divergence between pairs of duplicated IS elements.

To identify gene duplicates in *Acaryochloris* strains, clustering by *mmseqs* required coverage of at least 50% over the length of the target sequence, with a minimum amino acid identity of at least 50% over the length of the shorter sequence. Genes were annotated by comparing genes, using *DIAMOND BLASTp* v0.9.24.125 (Buchfink et al. 2015), with the annotated genome of *Acaryochloris* MBIC 11017 (Swingley et al. 2008) that is available in NCBI’s (National Center for Biotechnology Information) RefSeq database. Annotation data were also used to confirm the accuracy of gene clustering, where all members of each cluster of homologous genes are annotated with the same function.

RNASeq read data obtained for *A. marina* strains CCMEE 5410 and MBIC11017 (Gallagher and Miller 2018; NCBI SRA accession number PRJNA681975) were quality trimmed using Trimmomatic v0.39 (ILLUMINACLIP:TruSeq3-PE:2:30:10 LEADING:3 TRAILING:3 SLIDINGWINDOW:4:15 MINLEN:36) (Bolger et al. 2014). Given the heavy load of IS gene duplicates, including nearly identical duplicates, we performed read mapping using a custom approach that allowed us to keep accurate track of which reads map ambiguously. To estimate expression of genes present in multiple copies in each genome, a combination of *bowtie2* and *BLASTn*. *Bowtie2* v2.3.4.3 (default settings) (Langmead and Salzberg 2012) was used to recruit reads separately to each cluster of paralogous genes. To accurately estimate expression levels from each gene within each cluster, keeping track of ambiguously-mapping reads, the subset of reads mapping to each gene cluster was then queried against its respective gene cluster using BLASTn v2.9.0+ (qcov_hsp_perc = 100%, perc_identity = 100%) (Altschul et al. 1990). A custom Python script was then used to process the results and estimate *total* read counts from each gene cluster, as well unambiguous read counts from each *individual* gene within each cluster. Gene expression from single copy genes was estimated using only *bowtie2* (default settings), and the read count estimates were generated using *htseq-count* v0.11.2 (Anders et al. 2015). Gene expression values were generated by normalizing the read count estimates to transcripts per million (TPM) (Wagner et al. 2012). The TPM values reported for each gene/gene cluster and each time point represent the mean and standard deviation from five replicates. Transcriptomes from each time point were assembled using the default settings in *Trinity* v2.8.4 software (Haas et al. 2013). All custom Python scripts are available in the Supplementary Material online.

### Mutation Detection

We used *breseq* v0.33.2 (Deatherage and Barrick 2014) to identify mutations and their frequencies in the experimental populations with the strain CCMEE 5410 ancestral genome assembly as reference. Illumina-sequence FASTQ data (NCBI SRA accession number PRJNA685729) were first quality trimmed using *Trimmomatic* v0.39 (ILLUMINACLIP:TruSeq3-PE:2:30:10 HEADCROP:15 CROP:135 SLIDINGWINDOW:4:20 MINLEN:135; Bolger et al. 2014). *breseq* analyses were performed in polymorphism mode with a mutation frequency detection cutoff of 2%. For each candidate mutation, we used Fisher’s exact tests to test for biased strand representation and Kolmogorov–Smirnov tests to evaluate whether bases supporting a mutation had lower quality scores than those supporting the reference. We also manually inspected the alignments of reads to the reference for candidate mutations.

### Growth Rate Experiment

Cells from the ancestral population and from populations evolved for 400 generations were revived from −80 °C in fresh FeBG11 flasks and grown under laboratory evolution conditions to provide inoculum for the growth assay. Cells were inoculated into triplicate flasks containing 150 ml of FeMBG-11 to a starting OD_750_ value of 0.001. Flasks were incubated under lab evolution conditions as above, and OD_750_ of 2 ml subsamples was measured every 48 h. Growth rates were estimated for the exponential and linear phases of growth with two-parameter exponential and linear models, respectively. All statistical models were analyzed with JMP version 14.2 (SAS Institute Inc.).

## Supplementary Material

Supplementary data are available at *Genome Biology and Evolution* online.

## Supplementary Material

evab245_Supplementary_Data
