# Supplementary material for: Bacterial Adaptation by a Transposition Burst of an Invading IS Element
Source: Genome Biol Evol. 2021 Nov 13;13(11):evab245. doi: 10.1093/gbe/evab245 (PMC8763236; doi:10.1093/gbe/evab245)
Supplement: evab245_Supplementary_Data [file evab245_Supplementary_Data.zip › Amarina Supplementary figure legends.docx]

**Supplementary figure legends**

**Figure S1.** Frequency distribution of the relative gene content (top panel) and absolute number (bottom panel) of transposase genes for 642 bacterial and archaeal genomes downloaded from the NCBI RefSeq database. The arrows indicate *A. marina* CCMEE 5410. Each genome was annotated with Prokka, which uses Prodigal for gene prediction and the ISfinder database to search for transposases. After annotation, the number of total genes and transposases in each genome were counted using a custom Python script.

**Figure S2.** Relative frequencies of different IS families in *Acaryochloris* and *Cyanothece* genomes. The total number of transposase genes in each genome are indicated in parentheses.

**Figure S3.** Frequency plot of synonymous nucleotide divergence (d*S*) among duplicated transposase gene copies in the *A. marina* CCMEE 5410 genome. The mean is indicated with the solid line.

**Figure S4.** Representative batch culture growth curve for *A. marina* CCMEE 5410 during laboratory evolution. Growth was monitored by the increase in optical density at 750 nm, which is proportional to cell density.

**Figure S5**. GC content of coding and intergenic regions of the *A. marina* strain CCMEE 5410 genome. Coding regions included all CDS, tRNA, rRNA, and tmRNA genes. Intergenic GC content was calculated only for those intergenic regions that are longer than 100bp.

**Figure S6**. Nucleotide sequence alignment of the ISAm1 element reconstructed transcript and gene copies in the *A. marina* strain CCMEE 5410 genome (labels are genome coordinates). The transcript sequence is identical to the single complete copy of the element (6:36060).

**Figure S7**. Relative frequencies of the ancestral Sbt allele (blue) and ISAm-1 insertion mediated mutations (see inset) in the eight populations during laboratory evolution. Inset: Location and frequencies of the four mutations in *sbtAB* detected during 400 generations of laboratory evolution. Shown is a 728 bp region of the CCMEE 5410 genome including the 3’ end of *sbtA*, intergenic DNA and *sbtB*.
