# Supplementary material for: Bacterial Adaptation by a Transposition Burst of an Invading IS Element
Source: Genome Biol Evol. 2021 Nov 13;13(11):evab245. doi: 10.1093/gbe/evab245 (PMC8763236; doi:10.1093/gbe/evab245)
Supplement: evab245_Supplementary_Data [file evab245_Supplementary_Data.zip › Supplementary_figures.pdf]

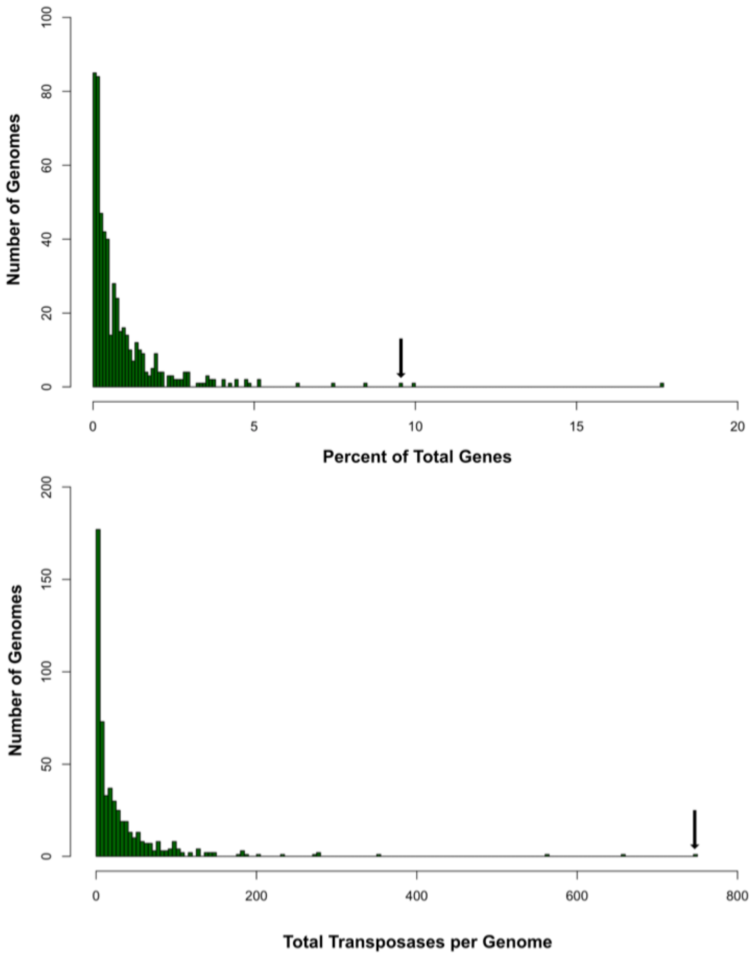

Fig.S1. Frequency distribution of the relative gene content (top panel) and absolute number (bottom panel) of transposase genes for 642 bacterial and archaeal genomes downloaded from the NCBI RefSeq database. The arrows indicate *A. marina* CCMEE 5410. Each genome was annotated with Prokka, which uses Prodigal for gene prediction and the ISfinder database to search for transposases. After annotation, the number of total genes and transposases in each genome were counted using a custom Python script.

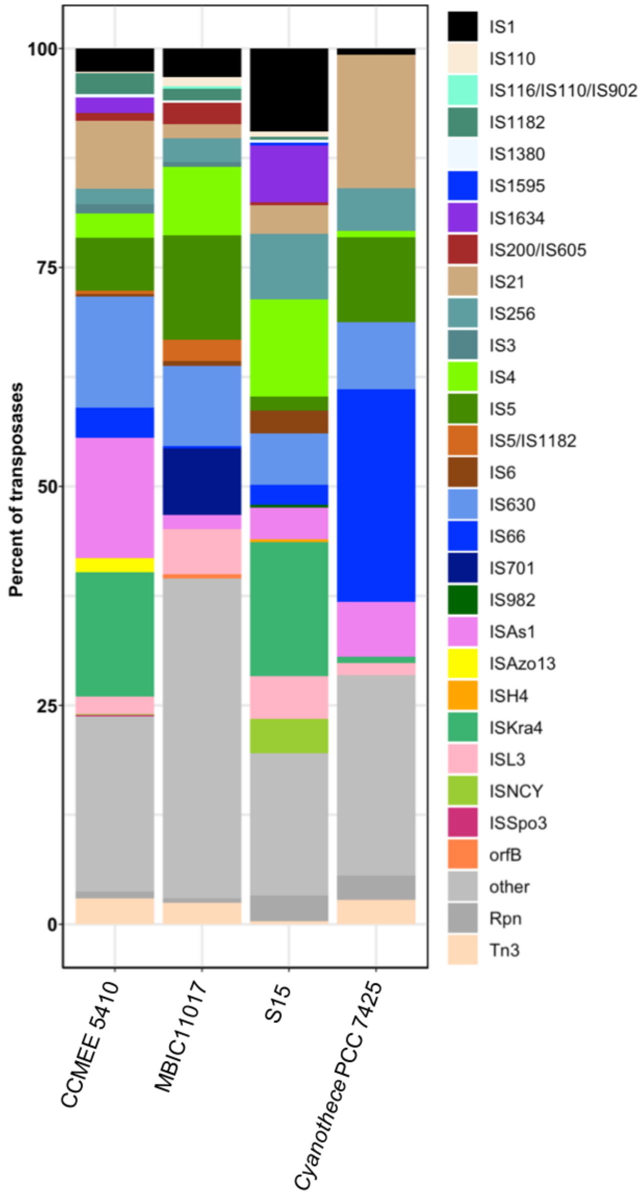

Fig. S2. Relative frequencies of different IS families in *Acaryochloris* and *Cyanothece* genomes.

A

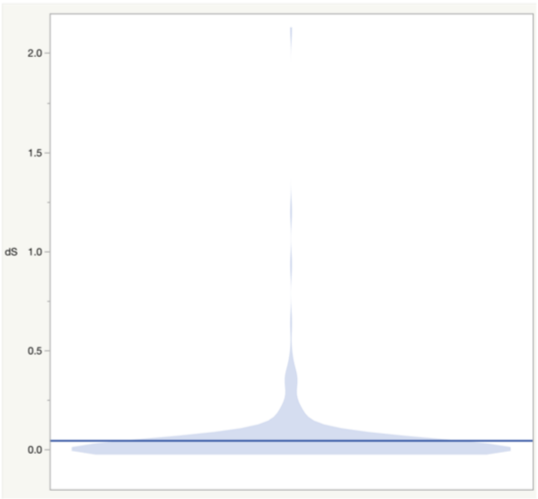

B

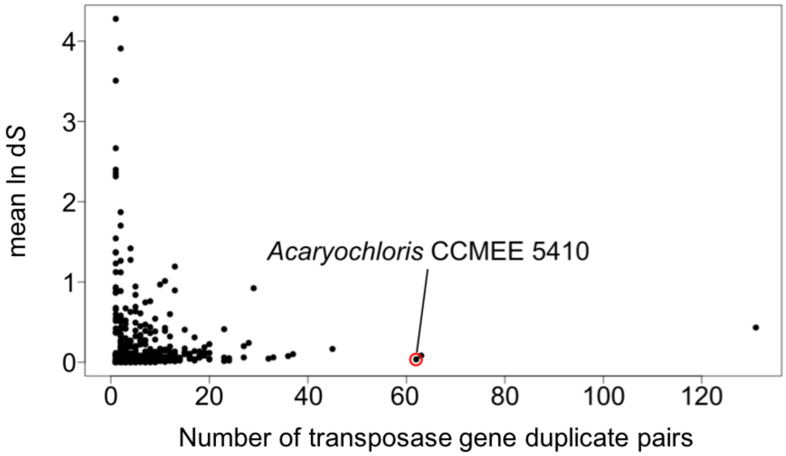

Fig.S3. A. Frequency plot of synonymous nucleotide divergence (d*S*) among duplicated transposase gene copies in the *A. marina* CCMEE 5410 genome. The mean is indicated with the solid line. **B.** Mean ln d*S* between transposase gene duplicates for 646 bacterial genomes downloaded from the RefSeq database.

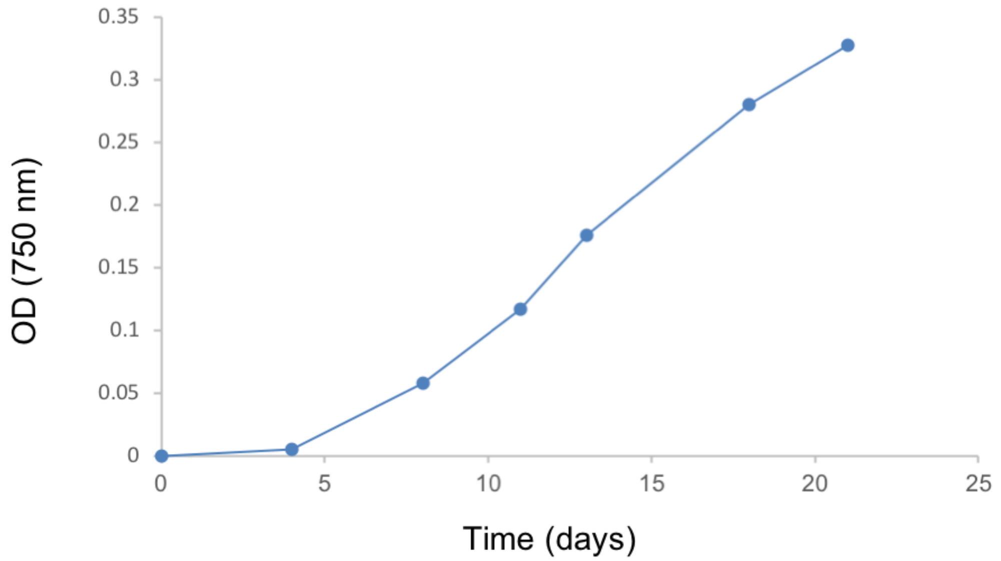

Fig.S4. Representative batch culture growth curve for *A. marina* CCMEE 5410 during laboratory evolution. Growth was monitored by the increase in optical density at 750 nm, which is proportional to cell density.

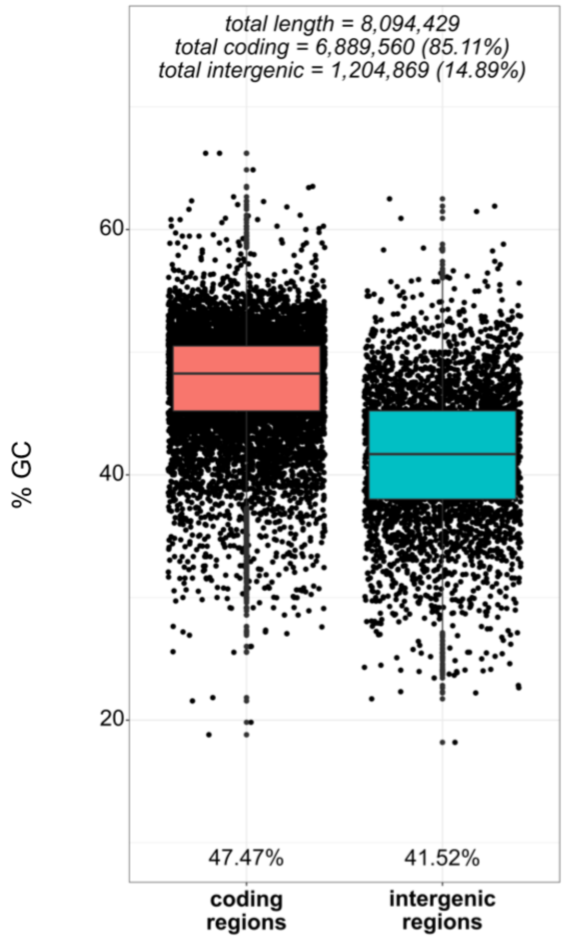

Fig. S5. GC content of coding and intergenic regions of the *A. marina* strain CCMEE 5410 genome. Coding regions included all CDS, tRNA, rRNA, and tmRNA genes. Intergenic GC content was calculated only for those intergenic regions that are longer than 100bp.

Fig. S6. Nucleotide sequence alignment of the ISAm1 element reconstructed transcript and gene copies in the *A. marina* strain CCMEE 5410 genome (labels are genome coordinates). The transcript sequence is identical to the single complete copy of the element (6:36060).

```
11:5896         ctggagtctggcaaaactgcttgattggccatcaaagaccacaccgtagtaaattcaggc     60
41:22642        ctggagtctggcaaaactgcttgattggccatcaaagaccacaccgtagtaaattcaggc     60
0:1118074       ctggagtctggcaaaactgcttgattggccatcaaagaccacaccgtagtaaattcaggc     60
0:4207749       ctggagtctggcaaaactgcttgattggccatcaaagaccacaccgtagtaaattcaggc     60
1:828563        ctggagtctggcaaaactgcttgattggccatcaaagaccacaccgtagtaaattcaggc     60
5:9073          ctggagtctggcaaaactgcttgattggccatcaaagaccacaccgtagtaaattcaggc     60
5:307000        ctggagtctggcaaaactgcttgattggccatcaaagaccacaccgtagtaaattcaggc     60
5:2970377       ctggagtctggcaaaactgcttgattggccatcaaagaccacaccgtagtaaattcaggc     60
6:36060         ctggagtctggcaaaactgcttgattggccatcaaagaccacaccgtagtaaattcaggc     60
mRNA            CTGGAGTCTGGCAAAACTGCTTGATTGGCCATCAAAGACCACACCGTAGTAAATTCAGGC     60
                ************************************************************

11:5896         cattaaccatcgtgtttgcccaaaggtgtagtcattctaaaaagcatcatccatccagac     120
41:22642        cattaaccatcgtgtttgcccaaaggtgtagtcattctaa-aagcatcatccatccagac     119
0:1118074       cattaaccatcgtgtttgcccaaaggtgtagtcattctaaaaagcatcatccatccagac     120
0:4207749       cattaaccatcgtgtttgcccaaaggtgtagtcattctaaaaagcatcatccatccagac     120
1:828563        cattaaccatcgtgtttgcccaaaggtgtagtcattctaaaaagcatcatccatccagac     120
5:9073          cattaaccatcgtgtttgcccaaaggtgtagtcattctaaaaagcatcatccatccagac     120
5:307000        cattaaccatcgtgtttgcccaaaggtgtagtcattctaaaaagcatcatccatccagac     120
5:2970377       cattaaccatcgtgtttgcccaaaggtgtagtcattctaaaaagcatcatccatccagac     120
6:36060         cattaaccatcgtgtttgcccaaaggtgtagtcattctaaaaagcatcatccatccagac     120
mRNA            CATTAACCATCGTGTTTGCCCAAAGGTGTAGTCATTCTAAAAAGCATCATCCATCCAGAC     120
                **************************************** *******************

11:5896         caggctctaaaacagtacctctcacacttgagcatcccgttatctaaacctcaacagcag     180
41:22642        caggctctaaaacagtacctctcacacttgagcatcccgttatctaaacctcaacagcag     179
0:1118074       caggctctaaaacagtacctctcacacttgagcatcccgttatctaaacctcaacagcag     180
0:4207749       caggctctaaaacagtacctctcacacttgagcatcccgttatctaaacctcaacagcag     180
1:828563        caggctctaaaacagtacctctcacacttgagcatcccgttatctaaacctcaacagcag     180
5:9073          caggctctaaaacagtacctctcacacttgagcatcccgttatctaaacctcaacagcag     180
5:307000        caggctctaaaacagtacctctcacacttgagcatcccgttatctaaacctcaacagcag     180
5:2970377       caggctctaaaacagtacctctcacacttgagcatcccgttatctaaacctcaacagcag     180
6:36060         caggctctaaaacagtacctctcacacttgagcatcccgttatctaaacctcaacagcag     180
mRNA            CAGGCTCTAAAACAGTACCTCTCACACTTGAGCATCCCGTTATCTAAACCTCAACAGCAG     180
                ************************************************************

11:5896         catgtgctgcgtattgttgaaggattgattgtgggcaatggccgcaaaaccccttagccac     240
41:22642        catgtgctgcgtattgttgaaggattgattgtgggcaatggccgcaa-accccttagccac     238
0:1118074       catgtgctgcgtattgttgaaggattgattgtgggcaatggccgcaaaaccccttagccac     240
0:4207749       catgtgctgcgtattgttgaaggattgattgtgggcaatggccgcaaaaccccttagccac     240
1:828563        catgtgctgcgtattgttgaaggattgattgtgggcaatggccgcaaaaccccttagccac     240
5:9073          catgtgctgcgtattgttgaaggattgattgtgggcaatggccgcaaaaccccttagccac     240
5:307000        catgtgctgcgtattgttgaaggattgattgtgggcaatggccgcaaaaccccttagccac     240
5:2970377       catgtgctgcgtattgttgaaggattgattgtgggcaatggccgcaaaaccccttagccac     240
6:36060         catgtgctgcgtattgttgaaggattgattgtgggcaatggccgcaaaaccccttagccac     240
mRNA            CATGTGCTGCGTATTGTTGAAGGATTGATTGTGGGCAATGGCCGCAAAACCCCTTAGCCAC     240
                *********************************************** ************

11:5896         ttgtatgctcagtgggttgatgctccagatgccagtgcagtggctgacttttttacgagtg     300
41:22642        ttgtatgctcagtgggttgatgctccagatgccagtgcagtggctgactt-ttacgagtg     297
0:1118074       ttgtatgctcagtgggttgatgctccagatgccagtgcagtggctgacttttttacgagtg     300
0:4207749       ttgtatgctcagtgggttgatgctccagatgccagtgcagtggctgacttttttacgagtg     300
1:828563        ttgtatgctcagtgggttgatgctccagatgccagtgcagtggctgacttttttacgagtg     300
5:9073          ttgtatgctcagtgggttgatgctccagatgccagtgcagtggctgacttttttacgagtg     300
5:307000        ttgtatgctcagtgggttgatgctccagatgccagtgcagtggctgacttttttacgagtg     300
5:2970377       ttgtatgctcagtgggttgatgctccagatgccagtgcagtggctgacttttttacgagtg     300
6:36060         ttgtatgctcagtgggttgatgctccagatgccagtgcagtggctgacttttttacgagtg     300
mRNA            TTGTATGCTCAGTGGGTTGATGCTCCAGATGCCAGTGCAGTGGCTGACTTTTTTACGAGTG     300
                ************************************************** *********
```

```
11:5896         agtacctggtctgagcaatctctcgacaaacgccttggggaaatc-acctggccgatgtc     359
41:22642        agtacctggtctgagcaatctctcgacaaacgccttggggaaatcaacctggccgatgtc     357
0:1118074       agtacctggtctgagcaatctctcgacaaacgccttggggaaatcaacctggccgatgtc     360
0:4207749       agtacctggtctgagcaatctctcgacaaacgccttggggaaatcaacctggccgatgtc     360
1:828563        agtacctggtctgagcaatctctcgacaaacgccttggggaaatcaacctggccgatgtc     360
5:9073          agtacctggtctgagcaatctctcgacaaacgccttggggaaatcaacctggccgatgtc     360
5:307000        agtacctggtctgagcaatctctcgacaaacgccttggggaaatcaacctggccgatgtc     360
5:2970377       agtacctggtctgagcaatctctcgacaaacgccttggggaaatcaacctggccgatgtc     360
6:36060         agtacctggtctgagcaatctctcgacaaacgccttggggaaatcaacctggccgatgtc     360
mRNA            AGTACCTGGTCTGAGCAATCTCTCGACAAACGCCTTGGGGAAATCAACCTGGCCGATGTC     360
                ********************************************* **************

11:5896         atagagcgcgtgcagcgaggaggaagttctcctgtggtgtatgtgagtattgatgactcg     419
41:22642        atagagcgcgtgcagcgaggaggaagttctcctgtggtgtatgtgagtattgatgactcg     417
0:1118074       atagagcgcgtgcagcgaggaggaagttctcctgtggtgtatgtgagtattgatgactcg     420
0:4207749       atagagcgcgtgcagcgaggaggaagttctcctgtggtgtatgtgagtattgatgactcg     420
1:828563        atagagcgcgtgcagcgaggaggaagttctcctgtggtgtatgtgagtattgatgactcg     420
5:9073          atagagcgcgtgcagcgaggaggaagttctcctgtggtgtatgtgagtattgatgactcg     420
5:307000        atagagcgcgtgcagcgaggaggaagttctcctgtggtgtatgtgagtattgatgactcg     420
5:2970377       atagagcgcgtgcagcgaggaggaagttctcctgtggtgtatgtgagtattgatgactcg     420
6:36060         atagagcgcgtgcagcgaggaggaagttctcctgtggtgtatgtgagtattgatgactcg     420
mRNA            ATAGAGCGCGTGCAGCGAGGAGGAAGTTCTCCTGTGGTGTATGTGAGTATTGATGACTCG     420
                ************************************************************

11:5896         accagtagcaaagataaggatacccatgccttggaaggggtggattggcagcatgaccac     479
41:22642        accagtagcaaagataaggatacccatgccttggaaggggtggattggcagcatgaccac     477
0:1118074       accagtagcaaagataaggatacccatgccttggaaggggtggattggcagcatgaccac     480
0:4207749       accagtagcaaagataaggatacccatgccttggaaggggtggattggcagcatgaccac     480
1:828563        accagtagcaaagataaggatacccatgccttggaaggggtggattggcagcatgaccac     480
5:9073          accagtagcaaagataaggatacccatgccttggaaggggtggattggcagcatgaccac     480
5:307000        accagtagcaaagataaggatacccatgccttggaaggggtggattggcagcatgaccac     480
5:2970377       accagtagcaaagataaggatacccatgccttggaaggggtggattggcagcatgaccac     480
6:36060         accagtagcaaagataaggatacccatgccttggaaggggtggattggcagcatgaccac     480
mRNA            ACCAGTAGCAAAGATAAGGATACCCATGCCTTGGAAGGGGTGGATTGGCAGCATGACCAC     480
                ************************************************************

11:5896         aatgccagtggtcgca-tactcccaagtacaagaaagggatggtgcatgtgagttgtcgg     538
41:22642        aatgccagtggtcgcaatactcccaagtacaagaaagggatggtgcatgtgagttgtcgg     537
0:1118074       aatgccagtggtcgcaatactcccaagtacaagaaagggatggtgcatgtgagttgtcgg     540
0:4207749       aatgccagtggtcgcaatactcccaagtacaagaaagggatggtgcatgtgagttgtcgg     540
1:828563        aatgccagtggtcgcaatactcccaagtacaagaaagggatggtgcatgtgagttgtcgg     540
5:9073          aatgccagtggtcgcaatactcccaagtacaagaaagggatggtgcatgtgagttgtcgg     540
5:307000        aatgccagtggtcgcaatactcccaagtacaagaaagggatggtgcatgtgagttgtcgg     540
5:2970377       aatgccagtggtcgcaatactcccaagtacaagaaagggatggtgcatgtgagttgtcgg     540
6:36060         aatgccagtggtcgcaatactcccaagtacaagaaagggatggtgcatgtgagttgtcgg     540
mRNA            AATGCCAGTGGTCGCAATACTCCCAAGTACAAGAAAGGGATGGTGCATGTGAGTTGTCGG     540
                **************** *******************************************

11:5896         gttcaaattggcaaccacagtgttcccttcgcctatcggctctatttacgggcaaaaacg     598
41:22642        gttcaaattggcaaccacagtgttcccttcgcctatcggctctatttacgggcaaaaacg     597
0:1118074       gttcaaattggcaaccacagtgttcccttcgcctatcggctctatttacgggcaaaaacg     600
0:4207749       gttcaaattggcaaccacagtgttcccttcgcctatcggctctatttacgggcaaaaacg     600
1:828563        gttcaaattggcaaccacagtgttcccttcgcctatcggctctatttacgggcaaaaacg     600
5:9073          gttcaaattggcaaccacagtgttcccttcgcctatcggctctatttacgggcaaaaacg     600
5:307000        gttcaaattggcaaccacagtgttcccttcgcctatcggctctatttacgggcaaaaacg     600
5:2970377       gttcaaattggcaaccacagtgttcccttcgcctatcggctctatttacgggcaaaaacg     600
6:36060         gttcaaattggcaaccacagtgttcccttcgcctatcggctctatttacgggcaaaaacg     600
mRNA            GTTCAAATTGGCAACCACAGTGTTCCCTTCGCCTATCGGCTCTATTTACGGGCAAAAACG     600
                ************************************************************

11:5896         gttcgcaacttgaaccggggacgtgccaaggaggagcgattgcgcttcca-accaagtat     657
41:22642        gttcgcaacttgaaccggggacgtgccaaggaggagcgattgcgcttccaaaccaagtat     657
0:1118074       gttcgcaacttgaaccggggacgtgccaaggaggagcgattgcgcttccaaaccaagtat     660
0:4207749       gttcgcaacttgaaccggggacgtgccaaggaggagcgattgcgcttccaaaccaagtat     660
```

```
1:828563      gttcgcaacttgaaccggggacgtgccaaggaggagcgattgcgcttccaaaccaagtat     660
5:9073        gttcgcaacttgaaccggggacgtgccaaggaggagcgattgcgcttccaaaccaagtat     660
5:307000      gttcgcaacttgaaccggggacgtgccaaggaggagcgattgcgcttccaaaccaagtat     660
5:2970377     gttcgcaacttgaaccggggacgtgccaaggaggagcgattgcgcttccaaaccaagtat     660
6:36060       gttcgcaacttgaaccggggacgtgccaaggaggagcgattgcgcttccaaaccaagtat     660
mRNA          GTTCGCAACTTGAACCGGGGACGTGCCAAGGAGGAGCGATTGCGCTTCCAAACCAAGTAT     660
              ************************************************** *********

11:5896       caactggtccgggagatgcttcagcagctccagcctctatacccaaagaat-ggc-gggt     715
41:22642      caactggtccgggagatgcttcagcagctccagcctctattacccaaagaatggcgggtg     717
0:1118074     caactggtccgggagatgcttcagcagctccagcctctattacccaaagaatggcgggtg     720
0:4207749     caactggtccgggagatgcttcagcagctccagcctctattacccaaagaatggcgggtg     720
1:828563      caactggtccgggagatgcttcagcagctccagcctctattacccaaagaatggcgggtg     720
5:9073        caactggtccgggagatgcttcagcagctccagcctctattacccaaagaatggcgggtg     720
5:307000      caactggtccgggagatgcttcagcagctccagcctctattacccaaagaatggcgggtg     720
5:2970377     caactggtccgggagatgcttcagcagctccagcctctattacccaaagaatggcgggtg     720
6:36060       caactggtccgggagatgcttcagcagctccagcctctattacccaaagaatggcgggtg     720
mRNA          CAACTGGTCCGGGAGATGCTTCAGCAGCTCCAGCCTCTATTACCCAAAGAATGGCGGGTG     720
              ****************************************  ** **  *  *** **

11:5896       gtacgtttattcgatagctggtatgcctccgccaaactactcaagtttgttcggcggcaa     775
41:22642      tacgttttattcgatagctggtatgcctccgccaaactactcaagtttgttcggcggcaa     777
0:1118074     tacgttttattcgatagctggtatgcctccgccaaactactcaagtttgttcggcggcaa     780
0:4207749     tacgttttattcgatagctggtatgcctccgccaaactactcaagtttgttcggcggcaa     780
1:828563      tacgt-ttattcgatagctggtatgcctccgccaaactactcaagtttgttcggcggcaa     779
5:9073        tacgttttattcgatagctggtatgcctccgccaaactactcaagtttgttcggcggcaa     780
5:307000      tacgttttattcgatagctggtatgcctccgccaaactactcaagtttgttcggcggcaa     780
5:2970377     tacgttttattcgatagctggtatgcctccgccaaactactcaagtttgttcggcggcaa     780
6:36060       tacgttttattcgatagctggtatgcctccgccaaactactcaagtttgttcggcggcaa     780
mRNA          TACGTTTTATTCGATAGCTGGTATGCCTCCGCCAAACTACTCAAGTTTGTTCGGCGGCAA     780
                    ******************************************************

11:5896       ggcaagcgatggttttg-ttgggcgctatcaaatccaatcgcattcttgatggcaagcgt     834
41:22642      ggcaagcgatggttttgtttgggcgctatcaaatccaatcgcattcttgatggcaagcgt     837
0:1118074     ggcaagcgatggtt-tgtttgggcgctatcaaatccaatcgcattcttgatggcaagcgt     839
0:4207749     ggcaagcgatggttttgtttgggcgctatcaaatccaatcgcattcttgatggcaagcgt     840
1:828563      ggcaagcgatggttttgtttgggcgctatca-atccaatcgcattcttgatggcaagcgt     838
5:9073        ggcaagcgatggttttgtttgggcgctatcaaatccaatcgcattcttgatggcaagcgt     840
5:307000      ggcaagcgatggttttgtttgggcgctatcaaatccaatcgcattcttgatggcaagcgt     840
5:2970377     ggcaagcgatggttttgtttgggcgctatcaaatccaatcgcattcttgatggcaagcgt     840
6:36060       ggcaagcgatggttttgtttgggcgctatcaaatccaatcgcattcttgatggcaagcgt     840
mRNA          GGCAAGCGATGGTTTTGTTTGGGCGCTATCAAATCCAATCGCATTCTTGATGGCAAGCGT     840
              ************** ** ************* ****************************

11:5896       ctgagtcaatggaacaaagacctcaagcacaaacactacgactcagttgagttaaaaaca     894
41:22642      ctgagtcaatggaacaaagacctcaagcacaaacactacgactcagttgagttaaaaaca     897
0:1118074     ctgagtcaatggaacaaagacctcaagcacaaacactacgactcagttgagttaa-aaca     898
0:4207749     ctgagtcaatggaacaaagacctcaagcacaaacactacgactcagttgagttaa-aaca     899
1:828563      ctgagtcaatggaacaaagacctcaagcacaaacactacgactcagttgagttaaaaaca     898
5:9073        ctgagtcaatggaacaaagacctcaagcacaaacactacgactcagttgagttaaaaaca     900
5:307000      ctgagtcaatggaacaaagacctcaagcacaaacactacgactcagttgagttaaaaaca     900
5:2970377     ctgagtcaatggaacaaagacctcaagcacaaacactacgactcagttgagttaaaaaca     900
6:36060       ctgagtcaatggaacaaagacctcaagcacaaacactacgactcagttgagttaaaaaca     900
mRNA          CTGAGTCAATGGAACAAAGACCTCAAGCACAAACACTACGACTCAGTTGAGTTAAAAACA     900
              ******************************************************* ****

11:5896       gtgacaggctcaaagca-acctacctaacgcgctcgattacgggccgattaaatgaggtg     953
41:22642      gtgacaggctcaaagcacacctacctaacgcgctcgattacgggccgattaaatgaggtg     957
0:1118074     gtgacaggctcaaagcacacctacctaacgcgctcgattacgggccgattaaatgaggtg     958
0:4207749     gtgacaggctcaaagcacacctacctaacgcgctcgattacgggccgattaaatgaggtg     959
1:828563      gtgacaggctcaaagcacacctacctaacgcgctcgattacgggccgattaaatgaggtg     958
5:9073        gtgacaggctcaaagcacacctacctaacgcgctcgattacgggccgattaaatgaggtg     960
5:307000      gtgacaggctcaaagcacacctacctaacgcgctcgattacgggccgattaaatgaggtg     960
5:2970377     gtgacaggctcaaagcacacctacctaacgcgctcgattacgggccgattaaatgaggtg     960
6:36060       gtgacaggctcaaagcacacctacctaacgcgctcgattacgggccgattaaatgaggtg     960
```

```
mRNA            GTGACAGGCTCAAAGCACACCTACCTAACGCGCTCGATTACGGGCCGATTAAATGAGGTG    960
                ***************** ******************************************

11:5896         ccttttgacgtctgtgtggtcatctccaagcggcaccctcgggattctcacccgaagtat    1013
41:22642        ccttttgacgtctgtgtggtcatctccaagcggcaccctcgggattctcacccgaagtat    1017
0:1118074       ccttttgacgtctgtgtggtcatctccaagcggcaccctcgggattctcacccgaagtat    1018
0:4207749       ccttttgacgtctgtgtggtcatctccaagcggcaccctcgggattctcacccgaagtat    1019
1:828563        ccttttgacgtctgtgtggtcatctccaagcggcaccctcgggattctcacccgaagtat    1018
5:9073          ccttttgacgtctgtgtggtcatctccaagcggcaccctcgggattctcacccgaagtat    1020
5:307000        ccttttgacgtctgtgtggtcatctccaagcggcaccctcgggattctcacccgaagtat    1020
5:2970377       ccttttgacgtctgtgtggtcatctccaagcggcaccctcgggattctcacccgaagtat    1020
6:36060         ccttttgacgtctgtgtggtcatctccaagcggcaccctcgggattctcacccgaagtat    1020
mRNA            CCTTTTGACGTCTGTGTGGTCATCTCCAAGCGGCACCCTCGGGATTCTCACCCGAAGTAT    1020
                ************************************************************

11:5896         tacctgtgcacagacacctcattgtctgcggccaaaatactgaaacgctactcaaagcgc    1073
41:22642        tacctgtgcacagacacctcattgtctgcggcca-aatactgaaacgctactcaaagcgc    1076
0:1118074       tacctgtgcacagacacctcattgtctgcggccaaaatactgaaacgctactcaaagcgc    1078
0:4207749       tacctgtgcacagacacctcattgtctgcggccaaaatactgaaacgctactcaaagcgc    1079
1:828563        tacctgtgcacagacacctcattgtctgcggccaaaatactgaaacgctactcaaagcgc    1078
5:9073          tacctgtgcacagacacctcattgtctgcggccaaaatactgaaacgctactcaaagcgc    1080
5:307000        tacctgtgcacagacacctcattgtctgcggccaaaatactgaaacgctactcaaagcgc    1080
5:2970377       tacctgtgcacagacacctcattgtctgcggccaaaatactgaaacgctactcaaagcgc    1080
6:36060         tacctgtgcacagacacctcattgtctgcggccaaaatactgaaacgctactcaaagcgc    1080
mRNA            TACCTGTGCACAGACACCTCATTGTCTGCGGCCAAAATACTGAAACGCTACTCAAAGCGC    1080
                ********************************** *************************

11:5896         tggtccattga-acagattattggtatctcaagcaatgtttgggattgggggagtttcgc    1132
41:22642        tggtccattgaaacagattattggtatctcaagcaatgtttgggattgggggagtttcgc    1136
0:1118074       tggtccattgaaacagattattggtatctcaagcaatgtttgggattgggggagtttcgc    1138
0:4207749       tggtccattgaaacagattattggtatctcaagcaatgtttgggattg-gggagtttcgc    1138
1:828563        tggtccattgaaacagattattggtatctcaagcaatgtttgggattgggggagtttcgc    1138
5:9073          tggtccattgaaacagattattggtatctcaagcaatgtttgggattgggggagtttcgc    1140
5:307000        tggtccattgaaacagattattggtatctcaagcaatgtttgggattgggggagtttcgc    1140
5:2970377       tggtccattgaaacagattattggtatctcaagcaatgtttgggattgggggagtttcgc    1140
6:36060         tggtccattgaaacagattattggtatctcaagcaatgtttgggattgggggagtttcgc    1140
mRNA            TGGTCCATTGAAACAGATTATTGGTATCTCAAGCAATGTTTGGGATTGGGGGAGTTTCGC    1140
                *********** ************************************ ***********

11:5896         gtccaacactatgaagcgattcacaagtggtactctttggtgcatttagcgttgcatttt    1192
41:22642        gtccaacactatgaagcgattcacaagtggtactctttggtgcatttagcgttgcatt-t    1195
0:1118074       gtccaacactatgaagcgattcacaagtggtactctttggtgcatttagcgttgcatt-t    1197
0:4207749       gtccaacactatgaagcgattcacaagtggtactctttggtgcatttagcgttgcatttt    1198
1:828563        gtccaacactatgaagcgattcacaagtggtactctttggtgcatttagcgttgcatttt    1198
5:9073          gtccaacactatgaagcgattcacaagtggtactctttggtgcatttagcgttgcatt-t    1199
5:307000        gtccaacactatgaagcgattcacaagtggtactctttggtgcatttagcgttgcatt-t    1199
5:2970377       gtccaacactatgaagcgattcacaagtggtactctttggtgcatttagcgttgcatt-t    1199
6:36060         gtccaacactatgaagcgattcacaagtggtactctttggtgcatttagcgttgcatttt    1200
mRNA            GTCCAACACTATGAAGCGATTCACAAGTGGTACTCTTTGGTGCATTTAGCGTTGCATTTT    1200
                ********************************************************** *

11:5896         ttgtatgctcaactgcgctgttctcaacagagggatgatccattcatttcaattgcc-ca    1251
41:22642        ttgtatgctcaactgcgctgttctcaacagagggatgatccattcatttcaattgcccaa    1255
0:1118074       ttgtatgctcaactgcgctgttctcaacagagggatgatccattcatttcaattgcccaa    1257
0:4207749       ttgtatgctcaactgcgctgttctcaacagagggatgatccattcatttcaattgcccaa    1258
1:828563        ttgtatgctcaactgcgctgttctcaacagagggatgatccattcatttcaattgcccaa    1258
5:9073          ttgtatgctcaactgcgctgttctcaacagagggatgatccattcatttcaattgcccaa    1259
5:307000        ttgtatgctcaactgcgctgttctcaacagagggatgatccattcatttcaattgcccaa    1259
5:2970377       ttgtatgctcaactgcgctgttctcaacagagggatgatccattcatttcaattgcccaa    1259
6:36060         ttgtatgctcaactgcgctgttctcaacagagggatgatccattcatttcaattgcccaa    1260
mRNA            TTGTATGCTCAACTGCGCTGTTCTCAACAGAGGGATGATCCATTCATTTCAATTGCCCAA    1260
                *********************************************************  *

11:5896         gtgattgaacatcaccgacagcaacaggctcaagcggtcttaatggctgcttgtgagcag    1311
41:22642        gtgattgaacatcaccgacagcaacaggctcaagcggtcttaatggctgcttgtgagcag    1315
```

```
0:1118074      gtgattgaacatcaccgacagcaacaggctcaagcggtcttaatggctgcttgtgagcag     1317
0:4207749      gtgattgaacatcaccgacagcaacaggctcaagcggtcttaatggctgcttgtgagcag     1318
1:828563       gtgattgaacatcaccgacagcaacaggctcaagcggtcttaatggctgcttgtgagcag     1318
5:9073         gtgattgaacatcaccgacagcaacaggctcaagcggtcttaatggctgcttgtgagcag     1319
5:307000       gtgattgaacatcaccgacagcaacaggctcaagcggtcttaatggctgcttgtgagcag     1319
5:2970377      gtgattgaacatcaccgacagcaacaggctcaagcggtcttaatggctgcttgtgagcag     1319
6:36060        gtgattgaacatcaccgacagcaacaggctcaagcggtcttaatggctgcttgtgagcag     1320
mRNA           GTGATTGAACATCACCGACAGCAACAGGCTCAAGCGGTCTTAATGGCTGCTTGTGAGCAG     1320
               ************************************************************

11:5896        gccatcacggatggcaatacgcaaggagtcgtgaagcgcttcattctaccaactcggatt     1371
41:22642       gccatcacggatggcaatacgcaaggagtcgtgaagcgcttcattctaccaactcggatt     1375
0:1118074      gccatcacggatggcaatacgcaaggagtcgtgaagcgcttcattctaccaactcggatt     1377
0:4207749      gccatcacggatggcaatacgcaaggagtcgtgaagcgcttcattctaccaactcggatt     1378
1:828563       gccatcacggatggcaatacgcaaggagtcgtgaagcgcttcattctaccaactcggatt     1378
5:9073         gccatcacggatggcaatacgcaaggagtcgtgaagcgcttcattctaccaactcggatt     1379
5:307000       gccatcacggatggcaatacgcaaggagtcgtgaagcgcttcattctaccaactcggatt     1379
5:2970377      gccatcacggatggcaatacgcaaggagtcgtgaagcgcttcattctaccaactcggatt     1379
6:36060        gccatcacggatggcaatacgcaaggagtcgtgaagcgcttcattctaccaactcggatt     1380
mRNA           GCCATCACGGATGGCAATACGCAAGGAGTCGTGAAGCGCTTCATTCTACCAACTCGGATT     1380
               ************************************************************

11:5896        gcagcctaatggcttgagaaacattagctgaattcgagttactgctctgaggcacacact     1431
41:22642       gcagcctaatggcttgagaaacattagctgaattcgagttactgctctgaggcacacact     1435
0:1118074      gcagcctaatggcttgagaaacattagctgaattcgagttactgctctgaggcacacact     1437
0:4207749      gcagcctaatggcttgagaaacattagctgaattcgagttactgctctgaggcacacact     1438
1:828563       gcagcctaatggcttgagaaacattagctgaattcgagttactgctctgaggcacacact     1438
5:9073         gcagcctaatggcttgagaaacattagctgaattcgagttactgctctgaggcacacact     1439
5:307000       gcagcctaatggcttgagaaacattagctgaattcgagttactgctctgaggcacacact     1439
5:2970377      gcagcctaatggcttgagaaacattagctgaattcgagttactgctctgaggcacacact     1439
6:36060        gcagcctaatggcttgagaaacattagctgaattcgagttactgctctgaggcacacact     1440
mRNA           GCAGCCTAATGGCTTGAGAAACATTAGCTGAATTCGAGTTACTGCTCTGAGGCACACACT     1440
               ************************************************************

11:5896        tcagagtaggttaccgcctgttctggcggtaatgaaaggaggctgaactttgatgaaatt     1491
41:22642       tcagagtaggttaccgcctgttctggcggtaatgaaaggaggctgaactttgatgaaatt     1495
0:1118074      tcagagtaggttaccgcctgttctggcggtaatgaaaggaggctgaactttgatgaaatt     1497
0:4207749      tcagagtaggttaccgcctgttctggcggtaatgaaaggaggctgaactttgatgaaatt     1498
1:828563       tcagagtaggttaccgcctgttctggcggtaatgaaaggaggctgaactttgatgaaatt     1498
5:9073         tcagagtaggttaccgcctgttctggcggtaatgaaaggaggctgaactttgatgaaatt     1499
5:307000       tcagagtaggttaccgcctgttctggcggtaatgaaaggaggctgaactttgatgaaatt     1499
5:2970377      tcagagtaggttaccgcctgttctggcggtaatgaaaggaggctgaactttgatgaaatt     1499
6:36060        tcagagtaggttaccgcctgttctggcggtaatgaaaggaggctgaactttgatgaaatt     1500
mRNA           TCAGAGTAGGTTACCGCCTGTTCTGGCGGTAATGAAAGGAGGCTGAACTTTGATGAAATT     1500
               ************************************************************

11:5896        tgccagactccag     1504
41:22642       tgccagactccag     1508
0:1118074      tgccagactccag     1510
0:4207749      tgccagactccag     1511
1:828563       tgccagactccag     1511
5:9073         tgccagactccag     1512
5:307000       tgccagactccag     1512
5:2970377      tgccag-------     1505
6:36060        tgccagactccag     1513
mRNA           TGCCAGACTCCAG     1513
               ******
```

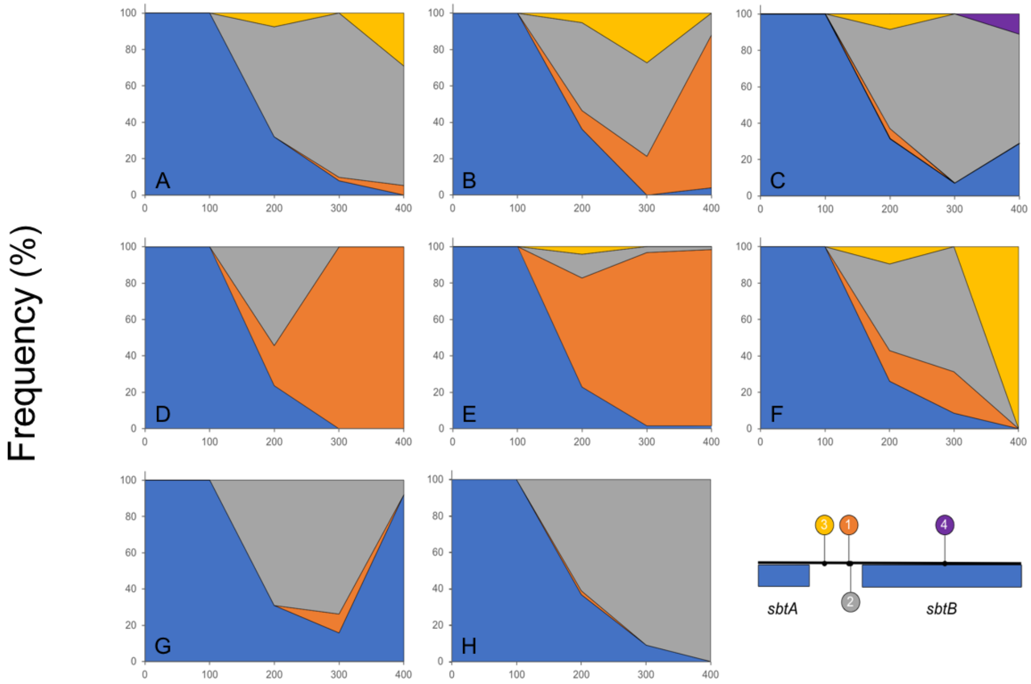

Fig. S7. Relative frequencies of the ancestral Sbt allele (blue) and ISAm-1 insertion mediated mutations (see inset) in the eight populations during laboratory evolution. Inset: Location and frequencies of the four mutations in *sbtAB* detected during 400 generations of laboratory evolution. Shown is a 728 bp region of the CCMEE 5410 genome including the 3' end of *sbtA*, intergenic DNA and *sbtB*.
